# Selected Papers from the 9th World Congress on Industrial Process Tomography

**DOI:** 10.3390/s19173804

**Published:** 2019-09-03

**Authors:** Manuchehr Soleimani, Thomas Wondrak, Chao Tan

**Affiliations:** 1 Engineering tomography lab (ETL), Department of Electronic & Electrical Engineering, University of Bath, Bath BA2 7AY, UK; 2 Helmholtz-Zentrum Dresden-Rossendorf, Institut für Fluiddynamik, Abt. Magnetohydrodynamik, 01328 Dresden, Germany; 3 School of Electrical and Information Engineering, Tianjin University, Tianjin 300072, China

Industrial process tomography (IPT) is a set of multi-dimensional sensor technologies and methods that aim to provide unparalleled internal information on industrial processes used in many sectors. The World Congress on Industrial Process Tomography (WCIPT) is the flagship conference of the International Society for Industrial Process Tomography (ISIPT. https://www.isipt.org/), which is held every two years. After successful previous events: UK (1999), Germany (2001), Canada (2003), Japan (2005), Norway (2007), China (2010), Poland (2013), and Brazil (2016), WCIPT-2018 took place in University of Bath, UK and WCIPT-2020 is planned to be hosted in Tianjin University in China. The field of IPT is gathering momentum and several large scale international projects such as EU Tomocon (https://www.tomocon.eu/) are well underway. 

A selection of papers was invited for this Special Issue. Of the 22 extended papers submitted to this Special Issue, 14 papers were accepted for publication. The authors in [1] introduce a new method for one-shot multi-frequency electrical tomography (ET). Promising early stage experimental results demonstrated that the method could indeed be useful in spectral imaging. In [2] the authors propose a fully automated software and design workflow for 3D electrical capacitance tomography (ECT) sensor, field modelling, and image reconstruction aided by 3D printers. Multilayer ECT sensors can be fabricated and linked to their corresponding computational and experimental system. In [3] the authors introduce a crowdsourcing approach with the example of highly complex silo flow imaging based on the X-ray tomography method. This provides a major step in the direction of human computer interfacing for IPT. In [4] a novel waste water metre is developed based on the ET method. The low cost ET system was verified with several experimental data with potential applications in environmental sustainable wastewater area. Authors in [5] show the use of electrical resistance tomography (ERT) for monitoring silica nanoparticles for sand-pack flooding experiments for oil recovery. The new ERT method has the potential to be used instead of X-ray CT or MRI for such an application offering a robust, online, and low cost solution. In [6] the authors demonstrate a novel image reconstruction method applied to magnetic induction tomography (MIT) for surface defect detection in metallic samples; the high resolution achieved in this approach makes MIT a unique candidate for such an application. The method was tested in lab-based experiments and was demonstrated for the first time using MIT data from a high temperature environment in continues casting in real steel manufacturing process. 

In [7] the author discusses the use of EIT for granular or solid materials. A correlation was demonstrated between the absolute impedance data and moisture content, an important parameter in many of these processes. Grouting ducts with steel strands are widely used to increase the structural strengths of infrastructures. The determination of the steel strand’s integrity inside of the ducts and the grouting quality are important for a strength evaluation of the structure. In [8], a capacitive sensing technique was applied to identify the cross-sectional distribution of the steel strands.

In [9] the authors present a new method of using a simulated inductor to help with capacitive coupled electrical impedance tomography (CCEIT). The new method of CCEIT offers great promise in the contactless imaging of complex dielectric properties, in particular, electrical conductivity, and this inductive compensation method is critical to enhancing the quality of the measured signals. In [10] a conformal mapping approach was developed for converting the open EIT geometry to the traditional circular EIT system. The method was verified using both simulated and real experimental data. The results greatly enhance our understanding of open EIT and planar array EIT. Calderon’s method has been successfully used for direct image reconstruction in ECT. In the method proposed in [11], the truncation radius adopted in the numerical integral greatly influences the reconstruction results. As our understanding of the complex value of ECT makes ECT a more versatile technology, the direct method proposed by the authors in [11], which directly reconstructs the admittivity, will become more important in near future. In [12], the authors introduce a new method to combine EIT and ultrasound tomography (UT). A Lagrange-Newton Method to combine EIT and UT reflection imaging a great step forward in realization of such a great multi-modality. In [13] the authors provide a comprehensive comparison of different machine learning (ML) methods for ET data. Similar to other IPT modalities the ET system are producing very large number of data in form of 2, 3, or 4D images. ML approaches will gain more and more traction as these imaging methods make their way into real industrial process applications. Wire-mesh sensors are used to determine the phase fraction of gas–liquid two-phase flow in many industrial applications. In [14], the authors report the use of a sensor to study the flow behaviour inside an offshore oil and gas industry device for subsea phase separation. 
